# Rheological and Mechanical Properties of Ultra-High-Performance Concrete Containing Fine Recycled Concrete Aggregates

**DOI:** 10.3390/ma12223717

**Published:** 2019-11-11

**Authors:** Lanzhen Yu, Lili Huang, Hui Ding

**Affiliations:** 1 Department of Architectural Engineering, Ningbo Polytechnic, Ningbo 315800, China; 0809102@nbpt.edu.cn; 2 The Faculty of Mechanical Engineering and Mechanics, Ningbo University, Ningbo 315211, China; dh570717457@163.com

**Keywords:** ultra-high-performance concrete, rheological properties, autogenous shrinkage, mechanical properties, pores structure, recycled concrete aggregates

## Abstract

The manufacturing process of ultra-high-performance concrete (UHPC) leads to a considerable amount of greenhouse gas emissions, which contribute to global warming. Using recycled concrete aggregates (RCA) to replace natural sand helps to reduce natural resources and energy consumption. In this study, the feasibility of manufacturing UHPC with fine RCA was investigated for the sustainable development of construction materials industry. We aimed to study the rheological properties, autogenous shrinkage, mechanical properties, and pore structure of UHPC with different amounts of RCA. The natural aggregate content was replaced with fine RCA at rates of 0, 20, 40, 60, 80, and 100 wt.%, and the packing density of the mixed fine aggregates in this study was estimated using a linear packing model. It was found that (1) the workability, mechanical properties, and deformation behaviour of UHPC with fine RCA were comparable to or even higher than those of UHPC made of high-quality aggregates; (2) the optimal replacement rate of fine RCA was in the range of 40–60 wt.%, considering the mechanical properties and deformation behaviour of UHPC; (3) the tensile strength, flexural strength, and Young’s modulus of UHPC increased by 6.18%, 12.82%, and 3.40%, respectively, when the replacement rate of fine RCA was 60 wt.%; (4) the maximum packing density of mixed fine aggregates presented a monotonic decreasing trend as the replacement percentage of fine RCA increased. These findings help to encourage and further promote the utilisation of RCA to produce UHPC.

## 1. Introduction

Ultra-high-performance concrete (UHPC) exhibits excellent mechanical properties and good durability, which have attracted considerable attention from scientific researchers and engineers. To achieve such high mechanical performance and good durability, manufacturers specifically design the mixture of UHPC to have a low water-to-binder ratio, a minimum pore size and steel fibres incorporated. Moreover, the raw materials used to produce UHPC are also of high standard, which is necessary to achieve its aforementioned excellent properties. However, the large amount of cement, steel fibre and high-quality aggregates (such as silica sand) not only raises the costs of UHPC but also depletes natural resources and causes environmental burden. There are generally three ways for UHPC to reach sustainable development: (1) reduce the cement content in it by using more supplementary cementitious materials, (2) reduce the natural aggregate content in it by using alternative aggregates, and (3) consume less cement in it by developing new construction techniques [1].

Natural aggregates (river sand and gravel), as important components of UHPC, take thousands of years to form, yet the consumption rate of these materials is much higher than their renewal rate. Natural aggregates are mostly used to produce construction and building materials [2]. In fact, river sand resources have experienced a tight supply situation in most countries around the world, especially in China. Moreover, critically, the excessive exploitation of natural river sand will inevitably cause desertification and degradation of the ecological environment [3]. Consequently, it is extremely necessary to find alternatives to these natural aggregates. Owing to the large-scale infrastructure construction in China, the construction and demolition (C&D) waste in the country reached 2.38 billion tons in 2017, which exceeded 30% of the municipal waste. Nevertheless, the utilisation coefficient of C&D waste in China is rather low (<10%) [4]. C&D waste often comes from concrete, bricks, ceramics, etc. Previously, C&D waste was crushed to manufacture recyclable aggregates, which are usually used as foundation materials. With the development of crushing and sorting technologies, mixed recycled aggregates can be used more efficiently because they can be made into fine recycled aggregates through further processing. Compared to the mechanical and physical properties of natural river sands, those of recycled sands are poorer because there are porous and patchy pieces in these sands. Therefore, when considering both the severe environmental impacts of and the urgent situation concerning C&D waste, it is necessary to determine whether the recycled concrete aggregates can be used to produce cementitious materials or not.

Currently, there are many previous studies that have assessed the effect of the different types and proportions of recycled concrete aggregates (RCA) obtained from C&D waste on the overall performance of recycled aggregate concrete (RAC). Brand et al. [5] indicated that the moisture content in coarse RCA has a contradictory impact on the fluidity of RAC. Kumar et al. [6] suggested that the workability of RAC is much less affected by the addition of 20 wt.% coarse and fine RCA if the added RCA is in saturated and surface dry condition. In addition, similar findings have also been confirmed by several other studies, which have indicated that the incorporation of RCA has little effect on the workability of RAC provided that there is no water consumption by the added RCA [7,8,9,10,11,12]. The mechanical properties (e.g., compressive strength, splitting tensile strength, flexural strength and Young’s modulus) of RAC generally decrease owing to the addition of RCA [13,14,15]. The compressive strength of RAC decreases by 20–50% when 100 wt.% RCA is used, as concluded by Exteberria et al. [16]. The result of the experimental investigation carried out by Thomas et al. [17] indicated that the splitting tensile strength of RAC decreases by 7–19% when 100 wt.% RCA is used. Verma and Ashish [18] evaluated the mechanical strength of RAC and pointed out that it is seriously affected if the replacement rate of RCA is more than 50%. Saha and Rajasekaran [19] made a similar conclusion that the mechanical strength of RAC obviously decreases when natural aggregates are replaced with coarse RCA. Tabsh and Abdelfatah [20] concluded that the mechanical strength of high-strength RAC (≥50 MPa) is almost not affected owing to the use of RCA. As for the Young’s modulus of RAC, Rao et al. [21] indicated that it decreases by 34.8% when 100 wt.% RCA is used. In the experimental findings of Xiao et al. [22], the decline rate for the Young’s modulus of RAC is 45% if the replacement rate of RCA is 100 wt.%. Limbachiya et al. [23] reported that the Young’s modulus of RAC is similar to that of regular concrete made with natural aggregates, supposing that the replacement rate of RCA is less than 30 wt.%. Although RCA have been extensively used to make normal concrete, self-consolidating concrete and high-strength concrete as mentioned above, their applications in UHPC, up to now, have remained limited. 

In general, the durability of RAC is poorer than that of regular concrete without RCA [13,14,24,25,26] because the adhered mortar on RCA has an adverse effect on the durability of RAC. The durability of RAC decreases with increasing amount of RCA, as concluded by Saravanakumar and Dhinakarn [27]. Pereira et al. [28] suggested that RAC is not suitable for use in aggressive environments. Thomas et al. [29] argued that the primary reason for the poor durability of RAC is its intrinsic porosity due to the use of RCA. Rao et al. [21] also found that the water absorption of RAC increases with increasing amounts of RCA. Compared to the resistance to chloride permeability of concrete without RCA, that of RAC decreases by 9.5% if the replacement rate of RCA is 100 wt.% [30]. Kanellopoulos et al. [31] also pointed out that the penetration coefficient of chloride ions for RAC increases with increasing amounts of RCA content. In addition, some studies are looking for ways to improve the durability of RAC [32,33,34,35]. It is thus clear that the mechanical properties and durability of normal RAC have been studied comprehensively in the literature. 

Thus far, both coarse and fine RCA can be used to produce regular concrete. In general, the specific surface area of fine RCA is larger than that of coarse RCA, and thus, the addition of fine RCA can result in a decrease in workability and mechanical strength for regular concrete [36]. As for UHPC, the situation may be different when fine RCA are used as a substitute for natural river sand because the workability of UHPC depends mainly on the efficiency of the water-reducing agent. The abovementioned negative effects on the workability of UHPC can be reduced significantly because of the high efficiency of the water-reducing agent. In addition, the high mechanical strength of UHPC is mainly due to its low water-to-binder ratio [37], which suggests that the strength of UHPC is also slightly affected by the use of fine RCA. Therefore, it may be feasible to manufacture UHPC by using fine RCA as a replacement for natural river sand. However, the use of fine RCA to produce UHPC has rarely been investigated in the literature, and thus, the performance of this type of UHPC needs to be systematically studied. Such an information gap necessitates further studies to gain an understanding of the subject.

In this study, we focused on investigating whether it is feasible to manufacture UHPC with fine RCA. On this basis, the rheological properties, autogenous shrinkage, mechanical properties (compressive strength, tensile strength, flexural strength and Young’s modulus) and pore structure of UHPC with different amounts of RCA were comprehensively investigated. The natural aggregate content was replaced with fine RCA at rates of 0, 20, 40, 60, 80 and 100 wt.%, and the packing density of the mixed fine aggregates was estimated through a linear packing model. The findings of this study provide an insight into the utility of fine RCA to manufacture UHPC.

## 2. Materials and Methods

### 2.1. Materials 

**Figure 1:**
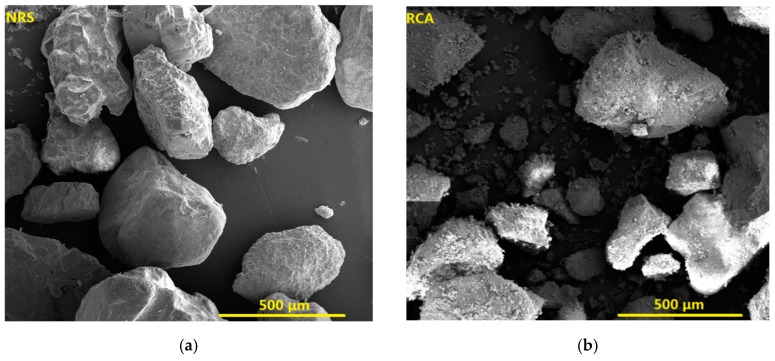
A micrograph of (**a**) NRS and (**b**) fine RCA.

**Figure 2:**
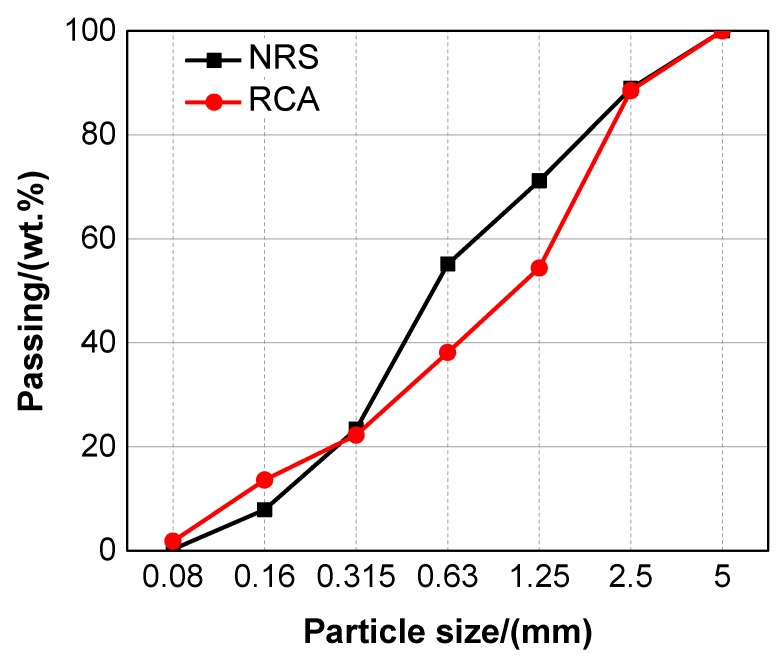
Sieve analysis of the natural river sand and fine RCA.

The cementitious materials used in the study contained P·II 52.5 cement, class I (equivalent to ASTM C 618 class F) fly ash and silica fume. The chemical compositions of these cementitious materials were characterised by X-ray fluorescence (XRF, ARL ADVANT’ XP, Thermo Fisher, Waltham, MA, USA) and are presented in Table 1. Natural river sand (NRS) was used as the fine aggregate, and its maximum size, specific gravity and water absorption were 5 mm, 2.65 and 1.27%, respectively. The fine RCA was derived from medium-strength concrete waste that was obtained from a dam in Yongjiang River in Ningbo, China. Moreover, the size, specific gravity and water absorption of the RCA were 0.06–5 mm, 2.61 and 6.24%, respectively. The preparation process of fine RCA was as follows: crushing, comminution by an impact crusher, magnetic separation and sieving by a double-deck screen. The fine RCA were pre-wetted to a saturated surface dry condition when they were used for mixing. A micrograph of the NRS and fine RCA is shown in Figure 1, and their particle size distributions are presented in Figure 2. A polycarboxylate-based high-range water reducer was used to obtain a satisfactorily workable UHPC. Its solid content, specific gravity and water-reducing rate were 34.0%, 1.07 and 31.6%, respectively. Straight steel fibres were used in the study, and their diameter, length and tensile strength were 0.20 mm, 13 mm and >3 GPa, respectively.

### 2.2. Mix Design

We are aiming to study the effects of the fine RCA content on the properties of UHPC, and thus, six different UHPC mixtures were designed in the work. The mixtures of UHPC used in the paper are shown in Table 2. The content of steel fibre in UHPC was 2.5 vol.%, and all the water-to-binder ratios of UHPC mixtures were set to 0.18. For convenience, the different mixtures were marked as N100R0, N80R20, N60R40, N40R60, N20R80 and N0R100, in which the N and R were the abbreviation for NRS and fine RCA, respectively. The initial mixture N100R0 indicated that the mixture contained 100 wt.% NRS and 0 wt.% fine RCA, and thus, it was the reference mixture.

The packing density of the aggregates plays an important role in the mix design of cement-based materials, and the overall performance of cement-based materials can be improved by optimising the packing density of the particles. The packing density of the aggregates can be calculated by some modelling methods. The packing density of the mixed fine aggregates in this study was estimated using a linear packing model. There are two basic assumptions in the model: 1) the aggregates are non-deformable and 2) the interactions among the aggregates are linear [2]. Assuming a mixture can be made of *n* items of size *d_x_* (*x* = 1, 2, 3 … *N*), we can order similar particles from the largest size to the smallest size or vice versa, with a known independent monosized packing density for each particle. The maximum packing density $\rho_{max}$ can be determined by the following equation:

(1) $$\rho_{max}=\min\left\{\frac{d_{x}}{1-\left(1-d_{x}\right)\sum_{y-1}^{x-1}f\left(x,\;y\right)\alpha_{y}-\sum_{y-x+1}^{n}l\left(x,\;y\right)\alpha_{y}}\right\}$$

where $d_{x}$ and $\alpha_{y}$ are the size packing density and the volume fraction of the *x*th component in the mixture, respectively, and the functions f(x, y) and l(x, y) denote the linear interactions contributed to by the smaller and larger particles originating from the filling and loosening effects in the particle packing, respectively. The symbol “min” represents the minimum value. In general, the monosized packing density maintains a constant value regardless of the boundary effect of the packing, and the value is taken as 0.63 according to the findings of Jiang et al. [2]. The functions f(x, y) and l(x, y) are expressed as follows:

(2) $$f\left(x,\;y\right)={\left(1-\frac{d_{y}}{d_{x}}\right)}^{2.0}+0.4\frac{d_{y}}{d_{x}}{\left(1-\frac{d_{y}}{d_{x}}\right)}^{3.7};\left(d_{y}\leq d_{x}\right)$$

(3) $$l\left(x,\;y\right)={\left(1-\frac{d_{x}}{d_{y}}\right)}^{3.3}+2.8\frac{d_{x}}{d_{y}}{\left(1-\frac{d_{x}}{d_{y}}\right)}^{2.7};\left(d_{y}>d_{x}\right)$$

The maximum packing density of the aggregates can be obtained via Equations (1)–(3).

### 2.3. Specimen Preparation

According to the above mix proportions, specimens of UHPC were cast without any mechanical vibration. The specimens were covered with plastic sheets after casting, cured for 48 h at room temperature and then demoulded. Subsequently, they were cured in a standard curing room with a temperature range of 20 ± 2 °C and a relative humidity of >95% for 28 days. 

### 2.4. Testing Methodologies

#### 2.4.1. Workability and Rheological Properties

The initial slump flow of the mortar of UHPC was maintained at 260 ± 5 mm to fulfil the requirement of a self-consolidating mortar. Hence, different amounts of high-range water reducer were used in the study. The slump flow was measured according to ASTM C 230/C 230M [38]. To study the rheological properties of UHPC, we used a co-axial viscometer (Viscometer 5, ConTec, Reykjavik, Iceland) to determine the yield stress and plastic viscosity of UHPC, according to the Bingham model [39]. The measurement of the rheological properties of UHPC was carried out in accordance with literature procedures [40]. As for these properties, the steel fibres were removed from the mix proportions of UHPC.

#### 2.4.2. Autogenous Shrinkage

According to ASTM C1698 [38], the autogenous shrinkage of UHPC was determined. The specimens were cast in corrugated plastic tubes (diameter: 5.8 ± 0.2 mm; length: 420 ± 5 mm), and then they were placed in a drying room with a temperature range of 22 ± 1 °C and a relative humidity of 50 ± 1%. The autogenous shrinkage of UHPC was measured every 12 h in the first day, thereafter, once a day for the first week and then once a week until the 28^th^ day.

#### 2.4.3. Mechanical Properties

According to the Chinese standard GB/T 50081-2002 [41], the compressive strength of UHPC was measured by using a 100 × 100 × 100 mm^3^ cubic specimen with a constant loading of 0.8 MPa/s. The flexural strength of UHPC was determined with a four-point bending loading configuration by using a 100 × 100 × 400 prism specimen, and the space between the loading points was one-third of the clear span, where there is no shear stress in the zone. In the flexural strength test, the specimens endured pure bending between two loading points at a distance of 100 mm, and the specimens were supported by two metal rollers, with the distance between them being 300 mm. The direct tensile strength of UHPC was measured on dog-bone specimens in accordance with literature procedures [42], and the details about the size information of the dog-bone specimens can also be found in Savino et al. [43]. The strain rate was 0.05 mm/min in the tensile test. Three replicated measurements were carried out on the mechanical properties of UHPC. The Young’s modulus of UHPC was measured using a universal testing machine and a micro-deformation testing facility, in which the experiment used a loading rate of 0.8 MPa/s, according to the Chinese standard GB/T 50081-2002 [41]. Moreover, the size of the specimens was 100 × 100 × 300 mm^3^ for the measurement of the Young’s modulus.

#### 2.4.4. Pore Structure

The porosity and pore size distribution of UHPC were tested by mercury intrusion porosimetry (MIP; Auto IV 9510, Micromeritics, Norcross, GA, USA), and the size of the sample used for MIP was about 5 × 5 × 5 mm^3^, which was split from the 100 × 100 × 100 mm^3^ cubic specimen. The hydration reaction of the UHPC sample used for MIP was terminated at 28th day by immersing the specimens in absolute alcohol. It should be pointed out that the samples were dried at 60 °C in a vacuum drying chamber for 72 h before the examination, and three replicated measurements were conducted for each UHPC mixture. In addition, an X-ray computed tomography (X-CT; MicroXCT-400, Xradia, Pleasanton, CA, USA) system and its corresponding analysis software (VG Studio MAX 3.0) were also used to investigate the pore structure of UHPC.

It should be pointed out that at least three repeated measurements were undertaken on the rheological properties, autogenous shrinkage, mechanical properties (compressive strength, tensile strength, flexural strength and Young’s modulus) and porosity of UHPC, to improve the accuracy of the experimental results. Moreover, only the mean values are reported in this paper.

## 3. Results and Discussion

### 3.1. Workability and Rheological Properties

The amount of high-range water reducer in UHPC mixtures could be used to assess the fluidity of UHPC since all the slump flow of the UHPC mixtures was maintained at 260 ± 5 mm in the study. The amount of high-range water reducer in different UHPC mixtures is given in Table 2. It can be seen from the table that the amount of high-range water reducer increased with the increase in the percentage of NRS replaced, which indicates that the workability of UHPC decreased with increasing amounts of fine RCA. This finding has been observed by other researchers [44,45,46]. The RCA consisted of porous and patchy pieces generated in the process of crushing concrete, and thus, there was adhered mortar on the surface of RCA, which caused greater internal friction during the mixing. In addition, the effectiveness of the high-range water reducer decreased because the specific surface area of fine RCA was higher than that of NRS [47]. Thus, the workability of UHPC decreased owing to the increased amount of fine RCA. 

**Figure 3:**
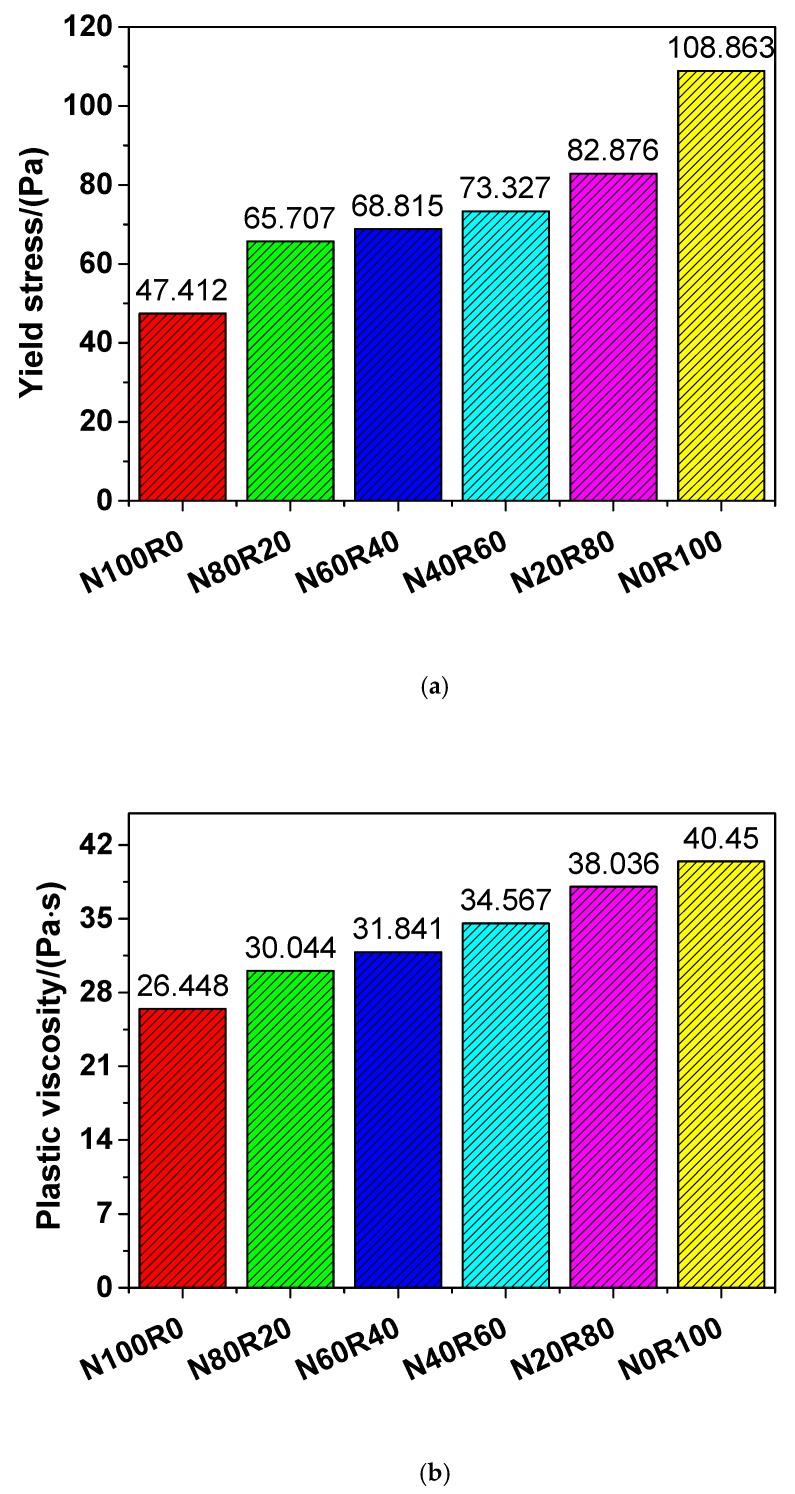
Rheological parameters of UHPC: (**a**) Yield stress and (**b**) Plastic viscosity.

According to the Bingham model, the yield stress and plastic viscosity of UHPC with different amounts of fine RCA were fitted, and the results are presented in Figure 3. As shown in the figure, both the yield stress and the plastic viscosity of UHPC increased with increasing amounts of fine RCA, which has also been observed in other studies [48,49]. The yield stress of N100R0, N80R20, N60R40, N40R60, N20R80 and N0R100 was 47.412, 65.707, 68.815, 73.327, 82.876 and 108.863 Pa, respectively, which suggests that the yield stress of UHPC increased by 38.59%, 45.14%, 54.66%, 74.80% and 129.61% when the natural aggregate content was replaced with fine RCA at rates of 20, 40, 60, 80 and 100 wt.%, respectively. The plastic viscosity of N100R0, N80R20, N60R40, N40R60, N20R80 and N0R100 was 26.448, 30.044, 31.841, 34.567, 38.036 and 40.450 Pa·s, respectively, which indicates that the plastic viscosity of UHPC rose by 13.60%, 20.39%, 30.70%, 43.81% and 52.94% when the natural aggregate content was replaced with fine RCA at rates of 20, 40, 60, 80 and 100 wt.%, respectively. As the replacement rates increased (i.e., 20, 40, 60, 80 and 100 wt.%), the inter-particle friction of UHPC increased, owing to the increased surface roughness of fine RCA, and thus, the yield stress of UHPC increased with increasing amount of fine RCA. Furthermore, the fine RCA with a large specific surface area needed more water in the UHPC to wrap and wet their solid surface, which resulted in the formation of more networks or flocculation structures in the UHPC. Therefore, the plastic viscosity of UHPC also increased with increasing amounts of fine RCA.

### 3.2. Autogenous Shrinkage

**Figure 4:**
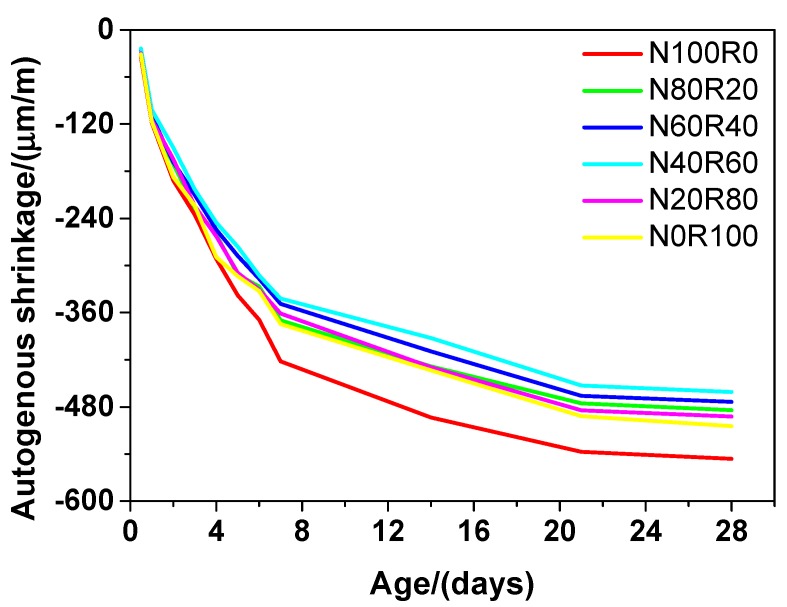
Autogenous shrinkage of UHPC.

The autogenous shrinkage of UHPC on the 28^th^ day is presented in Figure 4. It is interesting to note that the autogenous shrinkage of UHPC generally decreased because of the addition of fine RCA. The number of meso-pores in UHPC decreased owing to the addition of fine RCA, and thus, the autogenous shrinkage of UHPC caused by self-desiccation [40] decreased. On the whole, the autogenous shrinkage of UHPC followed the sequence N40R60 < N60R40 < N80R20 < N20R80 < N0R100 < N100R0, which suggests that when the natural aggregate content was replaced with fine RCA at a rate of 60 wt.%, the autogenous shrinkage of UHPC was lowest. For instance, the autogenous shrinkage of N40R60 and N100R0 on the 28^th^ day was 461.06 and 546.14 μm/m, respectively, which indicates that the former had a 15.58% lower autogenous shrinkage than that of the latter. What is more, the autogenous shrinkage of UHPC with NRS or fine RCA only (N100R0 and N0R100) was always higher than that of UHPC using mixed NRS and fine RCA (N20R80, N40R60, N60R40 and N80R20), which suggests that the autogenous shrinkage of UHPC could be decreased by using mixed fine aggregates. This could be mainly due to the increased porosity and pore size of UHPC owing to the use of mixed fine aggregates [50], which was also verified by the experiment results of this study concerning the porosity and pore size distribution of UHPC (see below).

### 3.3. Mechanical Properties

#### 3.3.1. Compressive Strength

**Figure 5:**
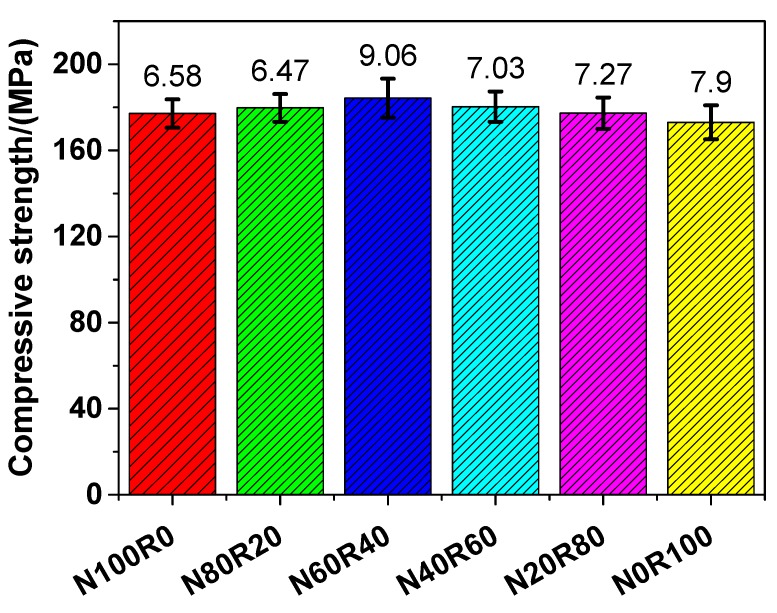
Compressive strength of UHPC.

The evolution of the compressive strength of the investigated UHPC is shown in Figure 5. The compressive strength of N100R0, N80R20, N60R40, N40R60, N20R80 and N0R100 was 177.13, 179.78, 184.26, 180.32, 177.30 and 173.05 MPa, respectively, which suggests that the compressive strength of UHPC with fine RCA (N80R20, N60R40, N40R60, N20R80 and N0R100) was similar to that of UHPC with NRS only (N100R0). As shown in Figure 5, there was a slight increase in the compressive strength of UHPC with fine RCA in comparison with N100R0, except for N0R100. When the replacement rate of fine RCA was 40 wt.%, the compressive strength of UHPC (N60R40) reached the maximum value, which shows that N60R40 had a 4.02% higher compressive strength than that of N100R0. The compressive strength of UHPC with fine RCA was between 173.05 and 184.26 MPa, which was comparable to that of UHPC manufactured with high-quality aggregates reported by other studies [40,51,52,53], and it was obviously higher than that of high-performance concrete with high quality silica sand [54,55,56,57].

In general, the compressive strength of UHPC is determined by three types of phases: mortar phase, aggregate phase, and the interface between these two. Compared to the other two phases, the aggregate phase is the strongest for UHPC with RCA, and thus, the quality of the aggregate has an important impact on the compressive strength of UHPC. Because there is some old mortar on the surface of RCA, the ITZ is weak in UHPC. The load is transmitted either via the ITZ or via the contacting points of the aggregates. There are three types of ITZ in UHPC with RCA: (1) the ITZ between the aggregate and the new cementitious paste, (2) the ITZ between the old cementitious paste and the new cementitious paste, and (3) the ITZ between the RCA and the old adhered mortar. Moreover, the third ITZ is the weakest because there are inherent micro-cracks in it and the stress concentration at the tips of the cracks is very high. The compressive strength of UHPC with fine RCA decreased owing to the appearance of a weak ITZ.

#### 3.3.2. Tensile Strength

**Figure 6:**
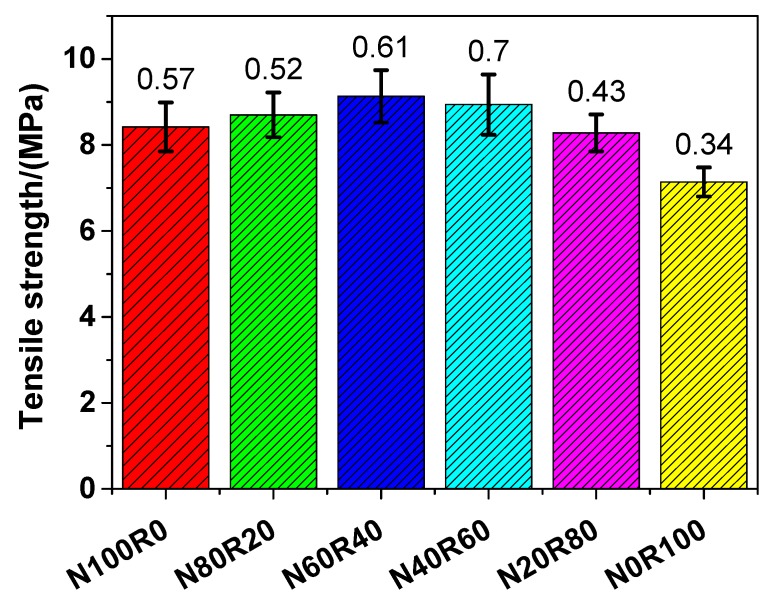
Tensile strength of UHPC.

The direct tensile strength of UHPC is presented Figure 6. The tensile strength of N100R0, N80R20, N60R40, N40R60, N20R80 and N0R100 was 8.42, 8.70, 9.13, 8.94, 8.28 and 7.14 MPa, respectively, which indicates that the tensile strength of UHPC with fine RCA (N80R20, N60R40, N40R60, N20R80 and N0R100) was similar to that of UHPC with NRS only (N100R0). The tensile strength of UHPC with fine RCA was higher than that of UHPC with NRS only when the replacement rate of fine RCA was lower than 60 wt.%. Like with the tensile strength of UHPC, when the replacement rate of fine RCA was 40 wt.%, the tensile strength of UHPC (N60R40) increased to the maximum value, which suggests that N60R40 had a 8.43% higher tensile strength than that of N100R0. The tensile strength of UHPC with fine RCA was in the range of 7.14–9.13 MPa, which was comparable to that of UHPC made of high-quality aggregates reported in the literature [58,59] and was slightly higher than that of UHPC with river sand and masonry sand [40]. As for the tensile strength of UHPC with fine RCA, there were two competing effects. On the one hand, the tensile strength of UHPC with RCA decreased because there were adhered porous mortars on the surface of RCA. On the other hand, the angularity of RCA could also improve the tensile strength of UHPC by formatting more mechanical bonds.

**Figure 7:**
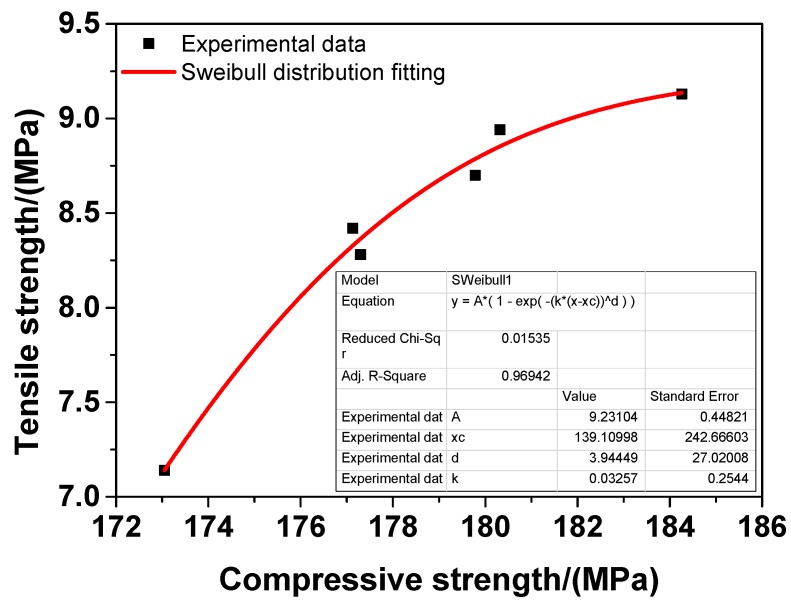
Relationship between tensile strength and compressive strength of UHPC.

In addition, the relationship between the direct tensile strength and the compressive strength of UHPC was investigated in the study and the results are presented in Figure 7. The direct tensile strength of UHPC increased monotonically with the increase in its compressive strength, as illustrated in Figure 7. The evolution of the direct tensile strength of UHPC was consistent, and the relationship between the direct tensile strength and the compressive strength of UHPC could be described by a Weibull distribution model, as shown by the fitting curve in Figure 7. The Weibull distribution model is expressed in detail as follows:

(4) $$\;\;\;\;\;F_{Ts}=9.23104\times \left\{1-\frac{1}{e^{\left[0.03257\times {\left(C-139.10998\right)}^{3.94449}\right]}}\right\}$$

where *F_Ts_* and C are the tensile strength and the compressive strength of UHPC, respectively.

The *R*-squared of the Weibull distribution fitting was 0.96942, which indicates that the fitting results agreed very well with the experimental results and that Equation (4) could be used to evaluate the direct tensile strength of UHPC by its compressive strength. Taken altogether, the Weibull distribution model established in the work was accurate enough to express the relationship between the direct tensile strength and the compressive strength of UHPC. In practice, the model could provide a simple way of characterising the tensile strength of UHPC via its compressive strength since the direct tensile strength of UHPC is generally much more difficult to obtain than its compressive strength. However, it should be noted that the empirical model might not accurately assess the direct tensile strength of UHPC with different mixtures (e.g., different water/binder ratios, different aggregate types and different types of steel fibres).

#### 3.3.3. Flexural Strength

**Figure 8:**
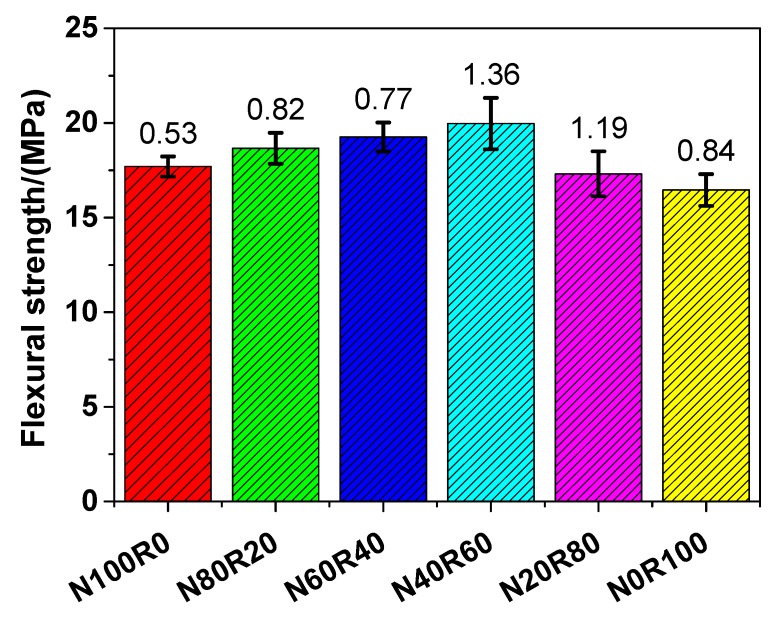
Four-point flexural strength of UHPC.

The variations in the four-point flexural strength of UHPC are illustrated in Figure 8. The four-point flexural strength of N100R0, N80R20, N60R40, N40R60, N20R80 and N0R100 was 17.70, 18.66, 19.26, 19.97, 17.31 and 16.46 MPa, respectively, which followed the sequence N40R60 > N60R40 > N80R20 > N100R0 > N20R80 > N0R100. This indicates that the four-point flexural strength of UHPC with fine RCA was higher than that of UHPC with NRS only when the replacement rate of fine RCA was lower than 60 wt.%. Unlike with the compressive strength and tensile strength of UHPC, when the replacement rate of fine RCA was 60 wt.%, the four-point flexural strength of UHPC (N40R60) reached the maximum value, which suggests that N40R60 had a 12.82% higher four-point flexural strength than that of N100R0. The four-point flexural strength of UHPC with fine RCA was in the range of 16.64–19.97 MPa, which was slightly higher than that of UHPC with river sand and nanoscale materials [60]. The flexural strength of UHPC was mainly affected by its specimen shape, surface roughness and Young’s modulus of the aggregates. The surface roughness of UHPC with RCA was rougher because of the adhered mortar on the surface of the specimen, and thus, the flexural strength of UHPC with RCA decreased. In addition, the Young’s modulus of RCA was lower than that of NRS, which also led to the decrease in flexural strength. Nevertheless, there were more mechanical bonds in the ITZ between RCA and the hydrated cement paste because of the angular shape of the RCA, which, in turn, contributed to the improvement in the flexural strength of UHPC with RCA.

#### 3.3.4. Young’s Modulus

**Figure 9:**
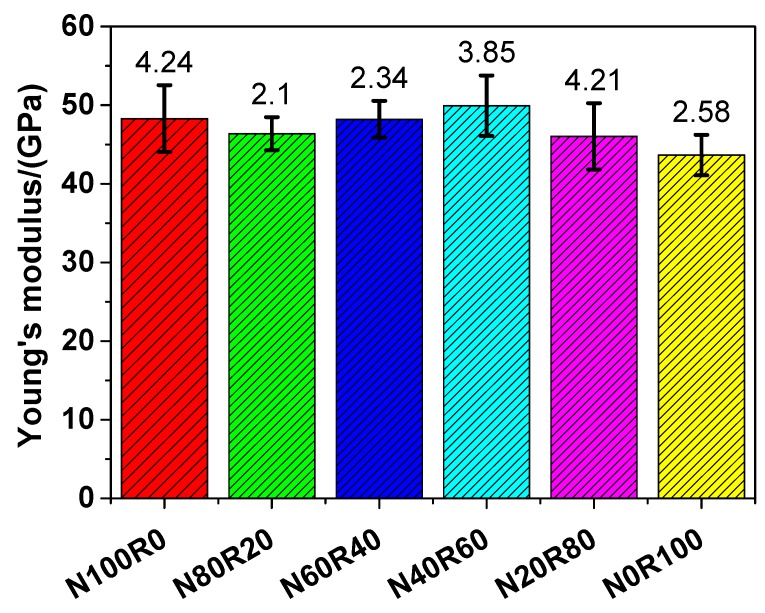
Young’s modulus of UHPC.

Figure 9 presents the Young’s modulus of UHPC. The Young’s modulus of N100R0, N80R20, N60R40, N40R60, N20R80 and N0R100 was 48.30, 46.38, 48.21, 49.94, 46.05 and 43.66 GPa, respectively, following the sequence N40R60 > N100R0 > N60R40 > N80R20 > N20R80 > N0R100, which suggests that the Young’s modulus of UHPC with fine RCA was lower than that of UHPC with NRS only, except for N40R60. Like with the four-point flexural strength of UHPC, when the replacement rate of fine RCA was 60 wt.%, the Young’s modulus of UHPC (N40R60) reached the maximum value, which indicates that N40R60 had a 3.40% higher Young’s modulus than that of N100R0. The Young’s modulus of UHPC with fine RCA was in the range of 43.66–49.94 GPa, which was slightly higher than that of UHPC with high-quality aggregates [61] and was comparable to that of UHPC made of recycled aggregates reported in the literature [4,62].

The Young’s modulus of UHPC was measured before the cracks initiated in it, and thus, the inherent micro-cracks in the ITZ are the key elements to the Young’s modulus of UHPC. The stiffness of UHPC with RCA decreased owing to the inherent micro-cracks during the preparation process of RCA and to the weak ITZ between the aggregate and the old adhered mortar, which led to the decrease in the Young’s modulus of UHPC with RCA. In addition, the stiffness of the mortar phase in UHPC with RCA could be reduced by the extra voids if there was more water during the hydration process, which also resulted in the decrease in the Young’s modulus of UHPC with RCA.

**Figure 10:**
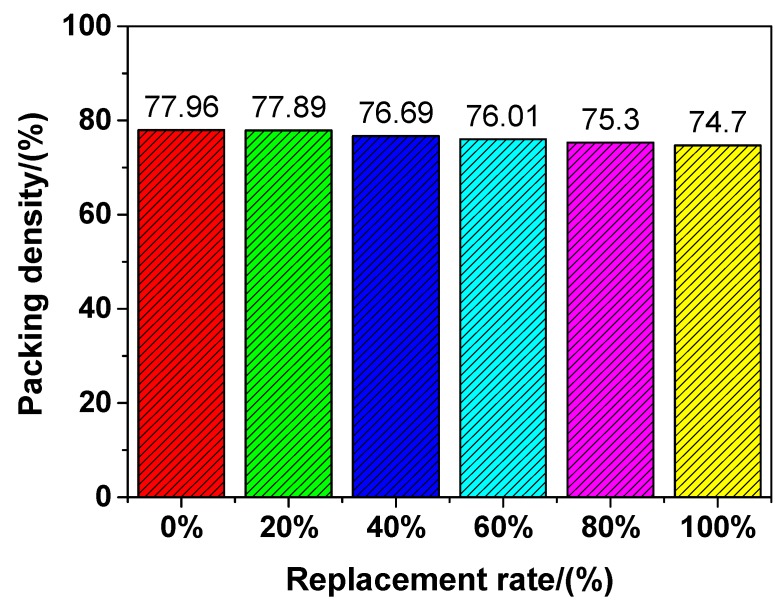
Maximum packing density of mixed fine aggregates.

According to Equations (1)–(3), the maximum packing density of mixed fine aggregates could be determined and the results are shown in Figure 10. The figure shows that it presents a monotonic decreasing trend as the replacement percentage of fine RCA increases. The lower packing density caused by the addition of fine RCA could have a negative impact on the properties of UHPC, which could be verified by the mechanical properties (see Figure 5, Figure 6 and Figure 8) of UHPC with fine RCA, which were lower than those of UHPC with NRS only when the replacement rate of fine RCA exceeded some value. However, the fine RCA contained approximately 2% very fine particles whose size is smaller than 0.08 mm, as shown in Figure 2. The very fine particles can result in a filling effect that can condense the packing of UHPC. The mechanical properties of UHPC could be improved by the rough-texture surface of fine RCA and by the filling effect of very fine RCA, and these two positive effects were not considered in the linear packing model mentioned above. These are the reasons why the mechanical properties of UHPC slightly improved owing to the addition of fine RCA.

### 3.4. Pore Structure

**Figure 11:**
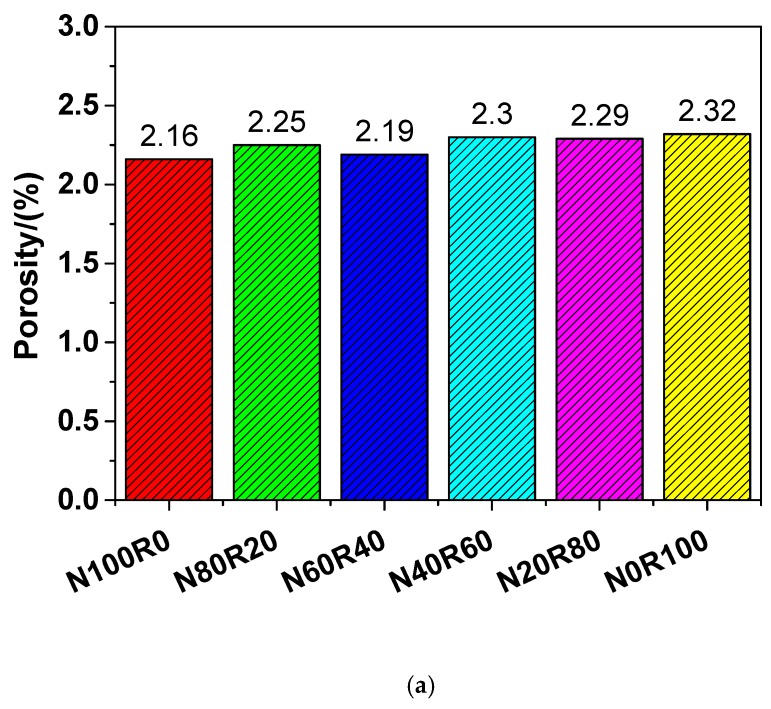

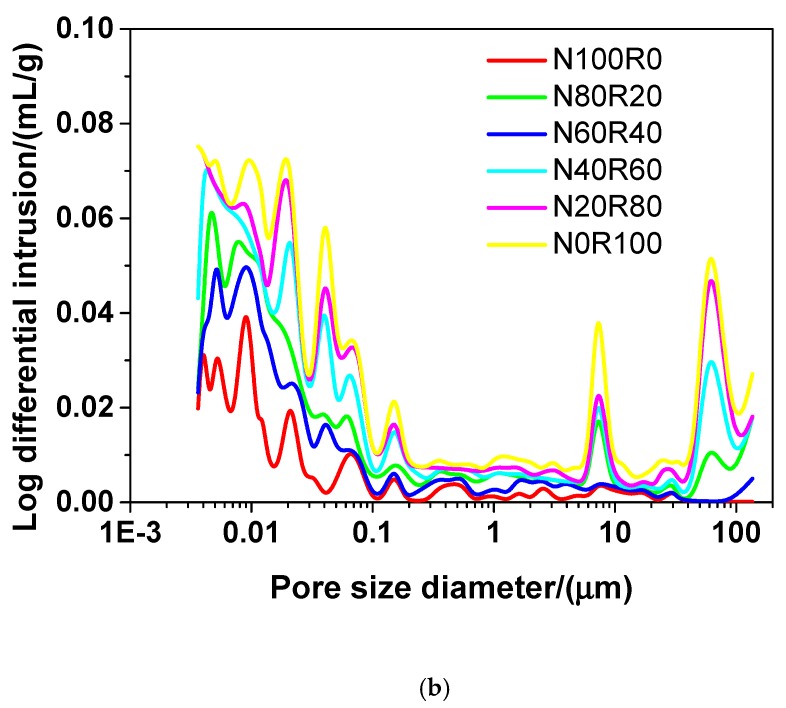
Porosity (**a**) and pore size distribution (**b**) of UHPC.

The porosity and pore size distribution of UHPC are summarised in Figure 11. The porosity of N100R0, N80R20, N60R40, N40R60, N20R80 and N0R100 was 2.16%, 2.25%, 2.19%, 2.30%, 2.29% and 2.32%, respectively, which followed the sequence N100R0 < N60R40 < N80R20 < N20R80 < N40R60 < N0R100. This indicates that the porosity of UHPC with fine RCA was slightly higher than that of UHPC with NRS only because the adhered mortar in RCA contained many micro-cracks during the crushing process of the old concrete and because of the presence of more ITZs owing to the addition of fine RCA. The porosity of UHPC basically increased with increasing amounts of fine RCA content, as illustrated in Figure 11a. When the replacement rate of fine RCA was 100 wt.%, the porosity of UHPC (N0R100) reached the maximum value, which suggests that N0R100 had a 7.41% higher porosity than that of N100R0.

As for the pore size distributions of N100R0, N80R20, N60R40, N40R60, N20R80 and N0R100, there were multi-peaks in the different types of UHPC, as shown in Figure 11b. Moreover, the changing trends in pore size diameter of the different types of UHPC were fairly close to each other. Therefore, the porosity and pore size diameter of UHPC increased owing to the use of fine RCA.

**Figure 12:**
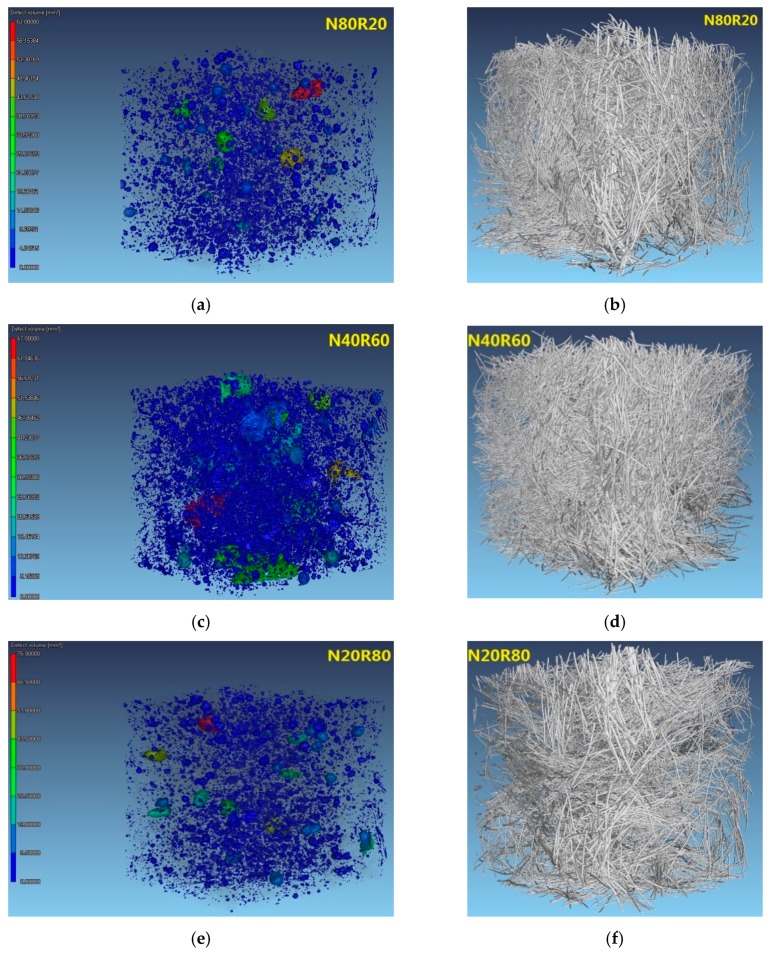
Pores and steel fibres distribution of UHPC by X-CT. (**a**) Pores distribution of N80R20; (**b**) Steel fibres distribution of N80R20; (**c**) Pores distribution of N40R60; (**d**) Steel fibres distribution of N40R60; (**e**) Pores distribution of N20R80; (**f**) Steel fibres distribution of N20R80.

In addition, the pore size and steel fibre distributions of UHPC were also studied by X-CT, and the results are presented in Figure 12. It is obvious from the figure that there were some large pores in the sample of UHPC because the sample was in a self-consolidating state without vibrating. The defect volume of N80R20, N40R60 and N20R80 was 63.00, 67.00 and 76.00 mm^3^, respectively, which was in good agreement with the result of their porosity. Furthermore, it can also be seen from Figure 12 that no steel fibre clusters were detected in the specimens of UHPC, which indicates that these fibres were dispersed uniformly in this study. Although the mixing and placing processes could have an impact on the isotropy of steel fibres, an isotropic and homogenous distribution of steel fibres was obtained through the special mixing procedure in this work.

In summary, the workability, mechanical properties and deformation behaviour of UHPC with fine RCA were comparable to or even higher than those of UHPC made of high-quality aggregates. Considering the mechanical properties and deformation behaviour of UHPC, we found the optimal replacement rate of fine RCA to be in the range of 40–60 wt.%. When the replacement rate of fine RCA was 60 wt.%, the UHPC containing fine RCA exhibited better performance on the 28^th^ day, with an autogenous shrinkage of 461.06 μm/m, compressive strength of 180.32 MPa, tensile strength of 8.94 MPa, flexural strength of 19.97 MPa, Young’s modulus of 49.94 GPa, and porosity of 2.39%. The findings of N40R60 mentioned above indicated that the comprehensive performance of UHPC was improved due the addition of fine RCA and that using fine RCA to produce UHPC contributed to reducing natural resource and energy consumption. It should be pointed out that the other important properties (e.g., dynamic mechanical properties, creep behaviour and durability aspects) of UHPC with fine RCA should be investigated comprehensively before using them in engineering applications. These findings help to encourage and further promote the use of RCA to produce UHPC.

## 4. Conclusions

The feasibility of manufacturing UHPC with fine RCA was investigated in this work. On this basis, the workability, deformation behaviour, mechanical properties and pore structure of UHPC with different amounts of RCA were systemically studied. In addition, the packing density of the mixed fine aggregates was estimated via a linear packing model. From the experimental results, the following important conclusions are drawn:

1. Both the yield stress and the plastic viscosity of UHPC increased with increasing amounts of fine RCA. When the natural aggregate content was replaced with fine RCA at a rate of 60 wt.%, the yield stress and the plastic viscosity of UHPC increased by 54.66% and 30.70%, respectively.
2. The autogenous shrinkage of UHPC generally decreased because of the addition of fine RCA and it was lowest when the replacement rate of fine RCA was 60 wt.%. The autogenous shrinkage of N40R60 and N100R0 after 28 days was 461.06 and 546.14 μm/m, respectively, with the former being 15.58% lower than the latter.
3. The compressive strength of UHPC with fine RCA was between 173.05 and 184.26 MPa, and it reached the maximum value when the replacement rate of fine RCA was 40 wt.%.
4. The tensile strength of UHPC with fine RCA was in the range of 7.14–9.13 MPa. When the replacement rate of fine RCA was 40 wt.%, the tensile strength of UHPC (N60R40) increased to the maximum value, which suggests that N60R40 had a 8.43% higher tensile strength than that of N100R0.
5. The relationship between the tensile strength and the compressive strength of UHPC could be described by a Weibull distribution model, and the model established in the work provided a simple way of characterising the tensile strength of UHPC via its compressive strength.
6. The four-point flexural strength of UHPC with fine RCA was in the range of 16.64–19.97 MPa. When the replacement rate of fine RCA was 60 wt.%, the four-point flexural strength of UHPC (N40R60) reached the maximum value, which suggests that N40R60 had a 12.82% higher four-point flexural strength than that of N100R0.
7. The Young’s modulus of UHPC with fine RCA was in the range of 43.66–49.94 GPa. When the replacement rate of fine RCA was 60 wt.%, the Young’s modulus of UHPC (N40R60) reached the maximum value, which indicates that N40R60 had a 3.40% higher Young’s modulus than that of N100R0.
8. According to the linear packing model established in the work, the maximum packing density of mixed fine aggregates displayed a monotonic decreasing trend when the replacement percentage of fine RCA was increased.
9. There were no steel fibre clusters detected in the specimens of UHPC, and an isotropic and homogenous distribution of steel fibres was obtained through the special mixing procedure in this work.
10. The findings of N40R60 concluded in the paper indicated that the comprehensive performance of UHPC was improved due the addition of fine RCA and that using fine RCA to produce UHPC contributed to reducing natural resource and energy consumption.

## Figures and Tables

**Table 1:** Chemical composition and physical properties of cement, silica fume, and fly ash.

Materials	Cement	Silica Fume	Fly Ash
Chemical Composition	(wt.%)		
CaO	63.50	0.85	8.07
SiO_2_	21.30	95.23	48.54
Al_2_O_3_	4.90	0.96	31.35
Fe_2_O_3_	3.52	0.79	5.24
MgO	0.95	0.81	2.53
SO_3_	1.84	1.36	1.27
K_2_O	0.82		1.58
Na_2_O			1.42
Loss on ignition	3.17		
Physical properties			
Specific gravity	3.15	2.24	2.55
Specific surface (m^2^/kg)	349.8	2.65 × 10^4^	
28d Compressive strength (MPa)	63.6		

**Table 2:** Mix proportions of UHPC (kg/m^3^).

Mixture	N100R0	N80R20	N60R40	N40R60	N20R80	N0R100
Cement	648	648	648	648	648	648
Fly ash	324	324	324	324	324	324
Silica fume	108	108	108	108	108	108
NRS	1188	950.4	712.8	475.2	237.6	0
RCA	0	237.6	475.2	712.8	950.4	1188
Water	194.4	194.4	194.4	194.4	194.4	194.4
Water reducer	21.6	21.6	21.7	21.9	22.0	22.3
Steel fiber	196	196	196	196	196	196
